# Tivantinib induces G2/M arrest and apoptosis by disrupting tubulin polymerization in hepatocellular carcinoma

**DOI:** 10.1186/s13046-015-0238-2

**Published:** 2015-10-12

**Authors:** Qingfeng Xiang, Zuojun Zhen, David YB Deng, Jingnan Wang, Yingjun Chen, Jieyuan Li, Yingfei Zhang, Fengjie Wang, Ningning Chen, Huanwei Chen, Yajin Chen

**Affiliations:** Department of Hepatopancreatobiliary Surgery, Sun Yat-sen Memorial Hospital, Sun Yat-sen University, Guangzhou, 510120 China; Department of Hepatopancreatobiliary Surgery, The First People’s Hospital of Foshan, Foshan, 528000 China; Research Center of Translational Medicine, The First Affiliated Hospital, Sun Yat-sen University, Guangzhou, 510080 China; Organ Transplant Center, The First Affiliated Hospital, Sun Yat-sen University, Guangzhou, 510080 China; Department of Spine Surgery, The First Affiliated Hospital, Sun Yat-sen University, Guangzhou, 510080 China

**Keywords:** Tivantinib, Hepatocellular carcinoma, MET, Cell cycle, Apoptosis

## Abstract

**Background:**

Tivantinib has been described as a highly selective inhibitor of MET and is currently in a phase III clinical trial for the treatment of hepatocellular carcinoma (HCC). However, the mechanism of tivantinib anti-tumor effect has been questioned by recent studies.

**Results:**

We show that tivantinib indiscriminately inhibited MET dependent and independent HCC cells proliferation. In contrast, other MET inhibitors, JNJ-38877605 and PHA-665752, just specifically inhibited the growth of MET dependent HCC cells. Tivantinib neither inhibit constitutive MET phosphorylation nor HGF-induced MET phosphorylation in HCC cells. In the microtubule polymerization analysis, tivantinib affected microtubule dynamics by a mechanism as a microtubule depolymerizer. Interesting, unlike other microtubule-targeting agents, paclitaxel and vincristine, tivantinib showed similar anti-proliferative activity in parental and multidrug-resistant cells. Further studies demonstrated that tivantinib induced a G2/M arrest and promoted apoptosis by both intrinsic and extrinsic pathway. The *in vivo* efficacy evaluation showed that tivantinib exhibited a good anti-tumor growth activity with anti-proliferative and pro-apoptotic effects.

**Conclusions:**

The potent anti-tumor activity of tivantinib in HCC was achieved by targeting microtubule. Tivantinib treatment for patients with HCC should not be selected based on MET status.

## Introduction

Hepatocellular carcinoma (HCC) is the third leading cause of cancer-related mortality globally, with standard chemotherapy being minimally effective in prolonging survival [1, 2]. While the approval of sorafenib was a significant step forward, the benefit is at best modest and confers a rather transient clinical benefit [3–5]. Moreover, in patients who are resistant, refractory or intolerant to sorafenib, no effective therapeutic options currently exist. Therefore, additional treatment options are warranted.

The *MET* proto-oncogene was originally identified as a fusion gene (tpr-met) in a chemically-transformed human osteosarcoma cell line [6]. MET is a high-affinity tyrosine kinase receptor (RTK) for hepatocyte growth factor (HGF). HGF binding triggers the dimerization of MET receptors, then activation of multiple intracellular pathways such as mitogen-activated protein kinase (MAPK), phosphatidylinositol 3-kinase (PI3K) and focal adhesion kinase (FAK) [7–9]. MET is overexpressed/activated in 20-40 % of HCC and correlated with poor prognosis [10, 11]. We and others have demonstrated that HCC cells with constitutive phosphorylation of MET (p-MET) is highly sensitive to MET kinase inhibitors *in vitro* and *in vivo* [12, 13]. In addition, MET activation triggered by anti-angiogenic therapies, such as sunitinib and sorafenib, can contribute to metastasis [12, 14–17]. Thus, it is conceivable that blockade of MET may be a promising therapeutic interventions in the treatment of HCC. Many anti-MET agents have been developed, some of which are ongoing clinical trials in HCC [18–20].

Tivantinib was first reported as a highly selective, orally administered, non-ATP competitive inhibitor of the MET, with an inhibitory constant (Ki) of 355 nM in biochemical assays [21]. The same work also showed that tivantinib inhibited constitutive p-MET and HGF-induced p-MET in several type of tumor cells with an IC_50_ of 100 to 300 nM. Tivantinib is being currently evaluated in the clinic as a specific MET inhibitor in different tumor types [22]. Results from phase II clinical trials showed that tivantinib increased a nearly doubling of progression-free survival (PFS) and overall survival (OS) in HCC patients with high expression of MET [22, 23]. More recently, a phase III clinical trial of tivantinib for the treatment of HCC was initiated. However, the mechanism of action of tivantinib have been questioned by recent studies. Two independent groups confirmed that tivantinib is an antimitotic agent that kills tumor cells independently of MET [24, 25]. Another study applying unbiased, mass-spectrometry spectrometry based, chemical proteomics approach, identified glycogen synthase kinase 3 (GSK3) alpha and beta as novel tivantinib targets [26]. Subsequent validation demonstrated that the anti-proliferation effect of tivantinib in non-small cell lung cancer (NSCLC) cells was mediated with its potent inhibition of GSK3α and β.

Although tivantinib is currently in a phase III clinical trial and has shown encouraging anti-tumor activity in HCC, the anti-tumor mechanism of tivantinib in HCC has not been fully elucidated. In particular, it is unclear whether tivantinib acts primarily through an anti-MET mechanism or whether it may also act to other targets as described above. Remarkably, anti-tumor agents that administered to patients before knowing their mechanism of action may be misleading in the development of predictive biomarkers. In the present study, we explored the molecular mechanism of anti-tumor activity of tivantinib in HCC.

## Materials and methods

### Chemicals and reagents

Tivantinib, JNJ-38877605 and PHA-665752 were purchased from Selleck Chemicals (Houston, TX, USA) and prepared as 20 mM stock solutions in DMSO (Sigma-Aldrich, St. Louis, MO, USA). For *in vivo* experiments, tivantinib and JNJ-38877605 were dissolved as previous described and administered via oral gavage at a dose of 10 ml/kg [21, 27]. Paclitaxel and vincristine were purchased from Sigma-Aldrich (St. Louis, MO). Primary antibodies against MET, phospho-MET (Tyr1234/1235), AKT, phospho-Akt (Ser473), ERK1/2, phospho-ERK1/2 (Thr202/Tyr204), α-tubulin, Cdc25C, Cylin B1, p21, FasL, Fas and horseradish peroxidase (HRP)-conjugated secondary antibodies were obtained from Cell Signaling Technology (Beverly, MA, USA). The antibody against Ki-67 was purchased from Dako (Santa Barbara, CA, USA). The cleaved caspase antibody sampler kit and GSK3 antibody sampler kit were purchased from Cell Signaling Technology (Beverly, MA). The antibody against GAPDH was a product of Kangchen Biotech (Shanghai, China). Cell Counting Kit-8 (CCK-8) was purchased from Dojindo Molecular Technologies Inc. (Kumamoto, Japan).

### Cell lines and culture conditions

Huh7, MHCC97L and MHCC97H cells were supplied by the Cell Bank of the Chinese Academy of Sciences (Shanghai, China). HepG2 and its doxorubicin (Dox) selected P-gp-overexpressing derivative HepG2/adr cells were kindly provided by Prof. Kwok-Pui Fung (The Chinese University of Hong Kong, Hong Kong) [28]. The human embryonic kidney HEK293 cell lines that were stably transfected with either *MDR1* (HEK293-*MDR1*) or the empty vector (HEK293-pcDNA3.1) were established by our laboratory [29]. These cell lines were maintained in DMEM (GIBCO BRL, Grand Island, NY, USA) supplemented with 10 % FBS (fetal bovine serum; GIBCO BRL, Grand Island, NY), 100 U/ml penicillin, and 100 U/ml streptomycin. All cell cultures were maintained at 37 °C in a CO_2_ incubator with a controlled humidified atmosphere composed of 95 % air and 5 % CO_2_.

### Cell proliferation assay

Cell proliferation was assessed using CCK-8 as previously reported [12]. Briefly, MHCC97L, MHCC97H, Huh7 and HepG2 cells (4–5 × 10^3^ per well) were plated in 96-well plates in DMEM supplemented with 10 % FBS and incubated at 37 °C, 5 % CO_2_. After 24 h, various concentrations of compounds were added into the wells, and the cells were incubated for another 72 h. Cells were then incubated for an additional 2 h with CCK-8 reagent (100 μl/ml medium) and read at 450 nm using a microplate reader (Thermo, Varioskan Flash, UAS). Each experiment was reproduced in six wells and repeated at least three times. The IC_50_ value, at which 50 % of cell growth inhibition compared with DMSO control, was calculated by nonlinear regression analysis using GraphPad Prism software (San Diego, CA).

### MET small interfering RNA (siRNA) and transfection

Synthetic siRNA specific for MET and non-targeting control siRNA were purchased from Santa Cruz Biotechnology (Santa Cruz, CA, USA). Cells were grown to 80 % confluence in 6-well plates and were transfected on the following day using Lipofectamine 2000 (LF2000; Invitrogen, Carlsbad, CA, USA). After 48 h of transfection, the expression of MET in transfected cells was verified by Western blot.

### Western blot analysis

Cell lysates were prepared as described previously [12]. A total of 25–50 μg of protein extracted from cultured cells was separated by SDS-PAGE and transferred onto PVDF membranes. The membranes were blocked and blotted with the relevant antibodies. HRP-conjugated secondary antibodies were detected using an enhanced chemiluminescence reagent (Millipore Corp., Bedford, MA, USA). GAPDH was used as a loading control. All the antibodies were used at a dilution of 1:1,000, except the anti-GAPDH antibody, which was used at a dilution of 1:5,000.

### Cell cycle analysis

Cells were plated at a density of 5 × 10^5^ in 6-well plates and then treated with either 0.1 % DMSO or various concentrations of tivantinib for 24 h. The cells were collected, fixed in 70 % ice-cold ethanol, and stored at 4 °C overnight. The samples were then incubated with 50 μg/ml RNase A for 30 min at 37 °C, followed by a 30 min treatment with 50 μg/ml propidium iodide (PI) at 37 °C. The fluorescence intensity of the stained cells was measured using flow cytometry (Beckman Coulter, USA). Data were analyzed using Flowjo software (San Carlos, California, USA).

### Apoptosis analysis

Apoptosis of tivantinib-induced HCC cells was assayed using an annexin V-FITC/PI staining kit (BD Biosciences San Diego, CA, USA). Following a 48 h treatment with 0.1 % DMSO or indicated concentrations of tivantinib, the cells were harvested, washed, and resuspended at a density of 5 × 10^5^/ml in 100 μl of 1× binding buffer. Annexin V-FITC and PI were then applied to each sample, and the reactions were incubated at room temperature for 15 min. Finally, another 400 μl of 1× binding buffer was added to each sample, and the samples were analysed using a flow cytometer (Beckman Coulter, Inc., Miami, FL, USA). The data were analysed using Kaluza software (Beckman Coulter, Inc.).

### Immunofluorescence staining

Cells were grown on glass coverslips in a 6-well culture plate until approximately 60 % confluent, and then were treated with tivantinib, vincristine or JNJ-38877605 for 24 h. The coverslips were fixed in 4 % paraformaldehyde for 15 min, followed by 0.5 % Triton X-100 for 5 min at room temperature. Fixed and permeabilized cells were blocked with 3 % BSA for 60 min at room temperature. Then, the cells were incubated with anti-α-tubulin (1:1000) as the primary antibody over night at 4 °C and Alexa Fluor 594-conjugated anti-rabbit IgG (1:1000) as the secondary antibody for 1 h at room temperature, DAPI was used for nuclear staining.

### Microtubule polymerization analysis

Microtubule polymerization assays were performed using a fluorescence-based tubulin polymerization assay kit (Cytoskeleton, Denver, CO, USA) according to the manufacturer’s instructions. Briefly, tubulin was re-suspended in ice cold G-PEM buffer (80 mM PIPES, 2 mM MgCl2, 0.5 mM EGTA, 1 mM GTP, 20 % (v/v) glycerol) and added to wells on a 96-well plate containing the designated concentration of experimental compounds. The paclitaxel and vincristine were used as polymer stabilizer and depolymerizer controls, respectively. Samples were mixed well and tubulin assembly was measured (emission wavelength is 420 nm; excitation wavelength is 360 nm) using a plate reader (DTX880, Beckman) at 37 °C and recorded every 60 s for 30 min.

### Animal experiments

#### Animal care

Female BALB/c athymic nude mice, 5 to 6 weeks old (Experimental Animal Center of Sun Yat-sen University, China), were used for *in vivo* studies. All animals were fed a standard diet ad libitum and housed in a temperature-controlled animal facility with a 12/12 h light/dark cycle. All procedures were performed according to the National Institutes of Health Guide for Care and Use of Laboratory Animals and were approved by the Bioethics Committee of Sun Yat-sen University.

#### Xenograft transplantation experiments

MHCC97L xenograft models were established by subcutaneous injection of tumor cells (5 × 10^7^/ml) in PBS with Matrigel at a 1:1 ratio. These cell suspensions were injected in a total volume of 0.2 ml into the right flank of each mouse and allowed to grow for approximately two weeks to reach a tumour size of roughly 80 to 200 mm^3^. The mice were then randomised into three groups (*n* = 6/group): Vehicle control (orally), tivantinib (100 mg/kg/d, orally) or tivantinib (200 mg/kg/d, orally). The tumor dimensions and body weights were measured every 3 days starting with the first day of treatment. The tumor volume (mm^3^) was calculated using the following formula: (l × w^2^)/2, where l and w refer to the larger and smaller dimensions collected at each measurement. The mice were sacrificed, and solid tumors were measured and excised after 15 days of treatment. The tumor growth inhibition (TGI) rate was calculated according to the following formula: (1 - *T*/*C*) × 100, where *T* indicates the mean weight of the tumor test groups and *C* indicates the mean tumor weight of the vehicle-treated group. To evaluate the effect of tivantinib on inhibition of MET expression *in vivo*, the xenografts established by MHCC97L cells were not initiated treatment until tumors reached 300 to 400 mm^3^ in size. The mice were then randomized into three groups (*n* = 3/group): ddH_2_O (orally), tivantinib (200 mg/kg/d, orally), or JNJ-38877605 (20 mg/kg/d, orally) for three days. The mice were sacrificed 3 h after the last treatment, and solid tumors were excised, split into two pieces, and either processed for paraffin embedding or homogenised in tumor lysis buffer for Western blot analysis.

#### Immunohistochemical analysis

To evaluate proliferation 5 μm paraffin-embedded sections were stained with anti-Ki67 (1:50) antibody. After blocking endogenous peroxidase activity, the sections were incubated overnight with the primary antibodies at 4 °C. Detection was completed with the Polink-2 Plus IHC Detection System (Beijing Zhongshan Biotechnology Co., Beijing, China) according to the manufacturer’s instructions. Sections were visualized by adding diaminobenzidine (DAB kit; Beijing ZhongShan Biotechnology Co.). Negative controls were obtained by omitting the primary antibody. Staining was evaluated by two independent observers. To quantify Ki-67 staining in sections, only nuclear immuno-reactivity was considered positive. The proliferation index corresponded to the number of labeled Ki-67 cells among at least 500 cells per region and are expressed as percentage.

#### TUNEL assay

The Apop Tag-Plus Kit (Millipore Corp.) was employed for the TUNEL assay according to the manufacturer’s instructions. Briefly, 5 μm paraffin-embedded sections were de-waxed and pretreated in 20 mg/ml proteinase K for 15 min at 37 °C. Subsequently, the sections were quenched in 3 % H_2_O_2_ at room temperature for 15 min and treated with terminal deoxynucleotidyl transferase (TdT) enzyme. The reaction was stopped by immersion in the stop/wash buffer for 15 min followed by PBS rinse for 10 min. The sections were subsequently incubated in anti-digoxigenin-peroxidase at room temperature for 30 min in a humidified chamber. After sufficient washing with PBS, the sections were visualized with DAB. Negative controls were incubated in medium lacking TdT enzyme. Staining was evaluated by two independent observers and only nuclear immuno-reactivity was considered positive. The apoptosis index corresponded to TUNEL-labelled cells among at least 500 cells per region and are expressed as percentage.

### Statistical analysis

Each experiment was performed at least three times, and the data are expressed as the mean ± standard deviation (SD). Significant differences were determined using Student's t-tests (two-tailed, α = 0.05).

## Results

### Tivantinib suppresses the growth of HCC cells was not due to inhibition of MET

**Fig. 1:**
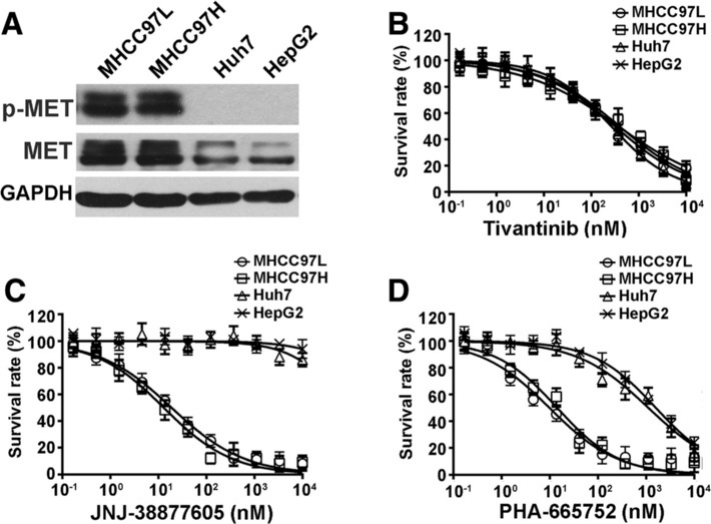
Tivantinib inhibits HCC cells proliferation independent of their MET status. **a** Western blot analysis was performed to detect the expression profile of MET in HCC cells. (**b**, **c** and **d**) Cytotoxicity assay. HCC cells were treated with different concentration of tivantinib, JNJ-38877605 and PHA-665752 for 72 h. IC_50_ was calculated by nonlinear regression analysis using GraphPad Prism software

Our previous study found that only HCC cells carrying constitutive p-MET expressing depend specifically on MET signaling for proliferation and survival, while impeding the MET pathway had no effect on the growth of p-MET negative HCC cells [12]. To evaluate if the anti-proliferative activity of tivantinib was due to inhibition of MET, we examined HCC cell lines that included two cell lines (MHCC97L and MHCC97H) with expression of p-MET and two HCC cells lines (Huh7 and HepG2) with negative expression of p-MET (Fig. 1a). As shown in Fig. 1b, the four HCC cell lines exhibited similar sensitivity to tivantinib. The IC_50_ values of tivantinib in MHCC97L, MHCC97H, Huh7 and HepG2 cells were 315 ± 26.3, 368 ± 45.4, 265 ± 18.7 and 392 ± 48.7 nM, respectively. Next, we compared the anti-proliferative activity of tivantinib with other MET inhibitors (JNJ-38877605 and PHA-665752). The data revealed that JNJ-38877605 and PHA-665752 selectively inhibited the growth of MHCC97L and MHCC97H cells without significantly affecting the proliferation of Huh7 and HepG2 cells (Fig. 1c and d). The IC_50_ values of JNJ-38877605 and PHA-665752 in Huh7 and HepG2 cells were more than 1000 nM.

### Tivantinib does not suppress constitutive p-MET and HGF-induced p-MET

**Fig. 2:**
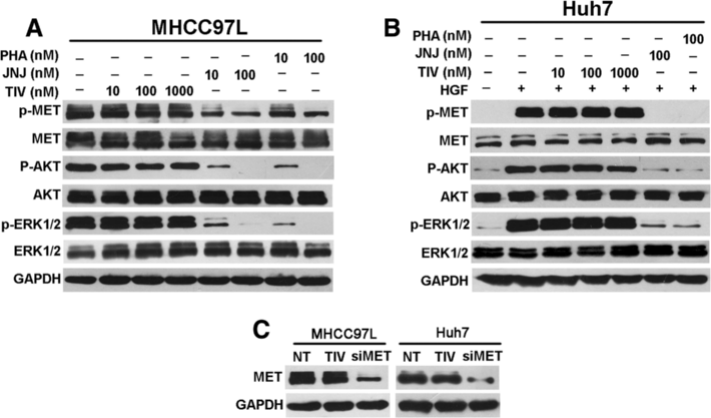
Effect of tivantinib on MET signaling in HCC cells. **a** Western blot analysis was conducted to measure effect of tivantinib on constitutive p-MET and downstream effectors in MHCC97L cells. Cells were treated with the indicated concentrations of tivantinib (TIV), JNJ-38877605 (JNJ) and PHA-665752 (PHA) in DMEM containing 10 % FBS for 4 h before protein extraction. **b** Western blot analysis was performed to detect effect of tivantinib on HGF-stimulated p-MET and downstream effectors in Huh7 cells. Cells were starved in medium containing 1 % FBS for 12 h before adding the indicated concentrations of compounds. After incubation for 4 h and stimulation with 50 ng/ml HGF for 10 min, cells were lysed for Western blot analysis. (**c**) MHCC97L and Huh7 cells were treated with 1000 nM tivantinib in DMEM containing 10 % FBS for 24 h before protein extraction. The MET siRNA-transduced cells were used as controls

We further selected MHCC97L and Huh7 cells to investigate the effect of tivantinib treatment on constitutive p-MET and HGF-induced p-MET *in vitro*, respectively. Marked suppression of p-MET was observed in MHCC97L cells tested after 4 h incubation with JNJ-38877605 and PHA-665752 at concentrations as low as 10 to 100 nM (Fig. 2a). Moreover, treatment with these doses also effectively abrogated the phosphorylation of downstream effectors, such as AKT and ERK1/2. In contrast, up to 1000 nM tivantinib had no detectable effect on the p-MET and its downstream pathway in MHCC97L cells (Fig. 2a). The ability of tivantinib to inhibit HGF-induced p-MET was analyzed in Huh7 cells. Not surprisingly, tivantinib failed to inhibit HGF- induced p-MET at any concentration tested (Fig. 2b). To examine the effect of tivantinib on the total MET expression, MHCC97L and Huh7 cells were treated with 1000 nM tivantinib for 24 h, MET siRNA-transduced cells were used as controls. As depicted in Fig. 2c, the expression of total MET did not affect by the treatment of tivantinib. The data presented above consistent with CCK8 results, collectively suggest that the effect of tivantinib on cell viability is not due to inhibition of MET.

### Tivantinib disturbs microtubule polymerization

**Fig. 3:**
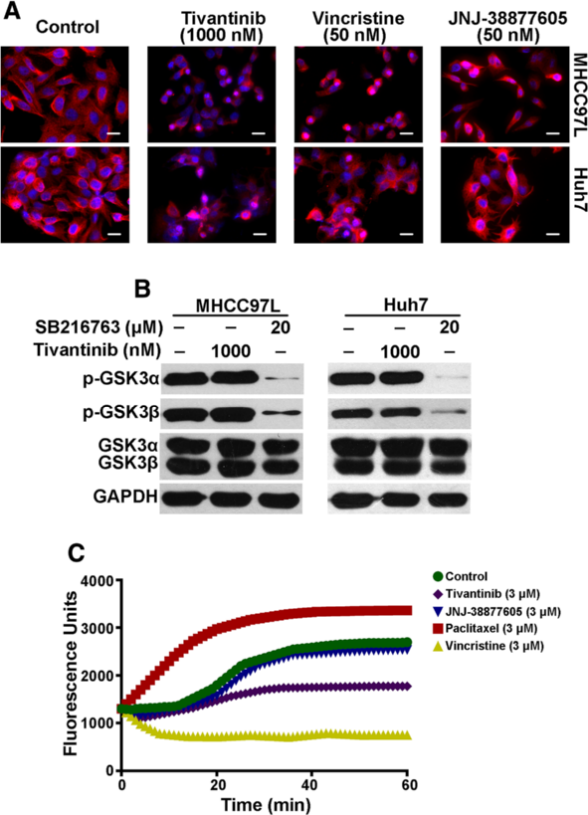
Tivantinib induces microtubules depolymerization in HCC cells independent of MET and GSK3. **a** MHCC97L and Huh7 cells were treated with tivantinib, vincristine or JNJ-38877605 for 12 h. After incubation, cells were reacted with monoclonal anti-a-tubulin antibody and Alexa Fluor 594-conjugated secondary antibody, DAPI was used for nuclear staining, the cellular microtubules were observed under fluorescence microscope. **b** MHCC97L and Huh7 cells were treated with tivantinib or SB216763 (a known GSK3 inhibitor) for 24 h. **c** Microtubule polymerization assay. Microtubules were incubated at 37°Cfor 1 h in the presence of tivantinib, paclitaxel (a microtubule polymer stabilizer), vincristine (a microtubule depolymerizer) or JNJ-38877605. Increased fluorescence indicates polymerization of microtubules. Scale bar, 20 μm

The works led by Basilico and Katayama indicated that tivantinib affected microtubule dynamics [24, 25]. To determine whether the anti-proliferation effect of tivantinib in HCC cells was achieved by disturbance microtubule dynamics. MHCC97L and Huh7 cells were treated with tivantinib, JNJ-38877605 and vincristine (a known microtubule depolymerizer agent) [30] for 12 h, and examined the effect on microtubules by immunofluorescent staining of α-tubulin. In both MHCC97L and Huh7 cells, treatment with tivantinib led to a loss of microtubules, similar to the vincristine treated cells (Fig. 3a). In contrast, the MET inhibitor JNJ-38877605 did not show any effect on microtubule stability in either cell lines. It has been reported that tivantinib inhibited the proliferation of NSCLC cells by targeting GSK3, which is a known inhibitor of microtubule stability [26]. Therefore, it is important to clarify whether anti-GSK3 activity are related to the effect of tivantinib on disruption of microtubule. Our Western blot analysis revealed that after exposure to 1000 nM tivantinib for 24 h, total and phosphorylated GSK3 α and β were not significantly changed in MHCC97L and Huh7 cells (Fig. 3b). We next evaluated whether tivantinib could directly affect microtubule stability applying a purified microtubule polymerization assay. Microtubules were incubated with experimental compounds at 37 °C for 60 min. Tivantinib treatment resulted in greatly inhibition of tubulin polymerization, whereas MET inhibitor JNJ-38877605 had no effect on tubulin polymerization (Fig. 3c). These results provide evidence that tivantinib disrupts microtubules in HCC cells by directly suppression of tubulin polymerization.

### Tivantinib showed equally anti-proliferative activity in P-glycoprotein (P-gp) overexpression multidrug resistance (MDR) cells

Although microtubule-targeting agents are highly effective anti-cancer agents and are extensively used in standard first-line chemotherapy, the development of resistance often renders them useless. P-gp is one of the ABC transporter proteins, which is frequently overexpressed in HCC [31, 32]. P-gp is known to facilitate the efflux of wildly used microtubule-targeting agents (taxanes and vinca alkaloids) and is attributed to the main obstacles to the success of chemotherapy for HCC [33]. For this reason, there is a strong need for new chemotherapeutic agents that could overcome P-gp mediated drug resistance. As illustrated in Table 1, the IC_50_ values of tivantinib in HepG2/adr, HepG2, HEK293-*MDR1* and HEK293-pcDNA3.1 cells were 412 ± 59.6, 392 ± 48.7, 327 ± 21.8 and 342 ± 35.6 nM, respectively. Tivantinib revealed cytotoxic activity equally against P-gp-overexpressed HepG2/adr and HEK293-*MDR1* cells compared with the effect on their parental cells suggesting tivantinib may not be a substrate of P-gp.

**Table 1:** Tivantinib overcome P-gp mediated MDR

Cell lines	Tivantinib IC_50_ (nM)	Paclitaxel IC_50_ (nM)	Vincristine IC_50_ (nM)
HepG2/adr	412 ± 59.6	5851 ± 52.4	3631 ± 67.8
HepG2	392 ± 48.7	15.6 ± 4.67	26.7 ± 6.23
HEK293-*MDR1*	327 ± 21.8	987 ± 43.8	1021 ± 92.3
HEK293-pcDNA3.1	342 ± 35.6	45.3 ± 8.54	17.1 ± 6.72

Cells were treated with different concentration of tivantinib for 72 h in DMEM containing 10 % FBS. IC_50_ was calculated by nonlinear regression analysis using GraphPad Prism software

### Tivantinib induces G2/M phase HCC cells

**Fig. 4:**
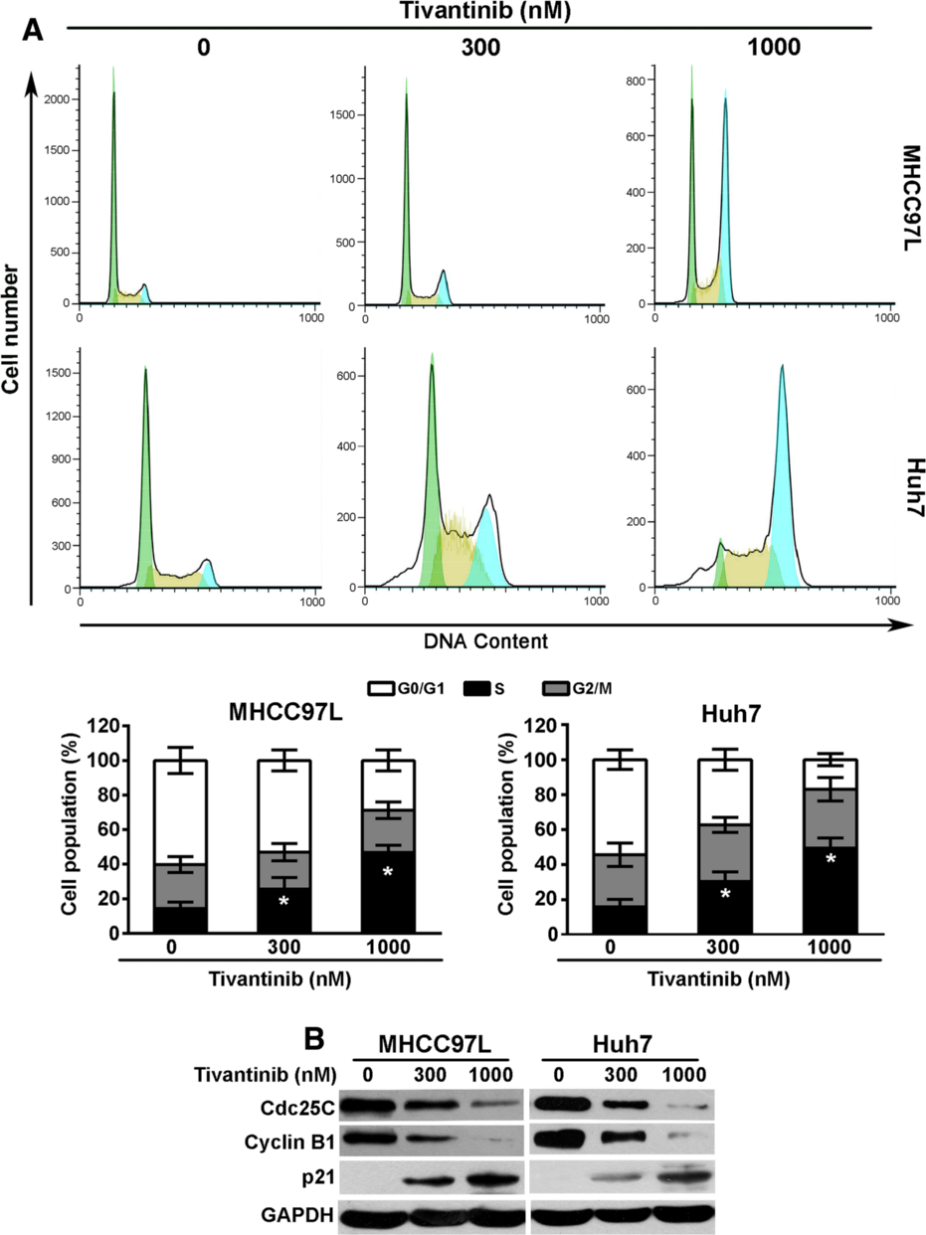
Tivantinib induces G2/M phase arrest and changes the expression of cell cycle related proteins in MHCC97L and Huh7 cells. **a** MHCC97L and Huh7 cells were treated with tivantinib for 24 h, and then cells were stained with propidium iodide (PI) for flow cytometric analysis. Data were analyzed using Flowjo software and were reported as the mean ± SD. **b** Changes of the expression of cell cycle related proteins in MHCC97L and Huh7 cells treated with tivantinib for 24 h. *, *P* < 0.05 vs. the control group

The above-mentioned data indicated that tubulin polymerization was interfered by tivantinib. Because most tubulin-targeting agents induce cell cycle arrest, it is expected that the influence of tivantinib on the cell cycle would be different from other MET inhibitors. To evaluate the cell cycle distribution of tivantinib-treated cells, flow cytometric analysis was conducted to analyze the cell cycle after treatment with various concentrations of tivantinib for 24 h. As shown in Fig. 4a, in both MHCC97L and Huh7 cells, tivantinib markedly increased the percentage of cells in the G2/M phase. Remarkably, previous studies demonstrated that specifically inhibit MET leading to profoundly G0/G1 arrest in MHCC97L cells by inhibition the expression of Cyclin D1 [12, 34].

To better understand the mechanisms of tivantinib-induced G2/M arrest, we examined the effect of tivantinib on the expression of proteins that regulate the G2/M transition. Cdc25C is a phosphatase which functions in the nucleus to trigger cell-cycle progression by activating the Cdc2-cyclin B mitotic kinase complex, thereby allowing cell entry into mitosis [35]. In MHCC97L and Huh7 cells, tivantinib treatment significantly down-regulated the expression of Cdc25C and cyclin B1. Furthermore, the G2–M checkpoint regulator p21 [36, 37] was remarkably increased following tivantinib treatment (Fig. 4b).

### Tivantinib induces apoptosis of HCC cells via intrinsic and extrinsic pathway

**Fig. 5:**
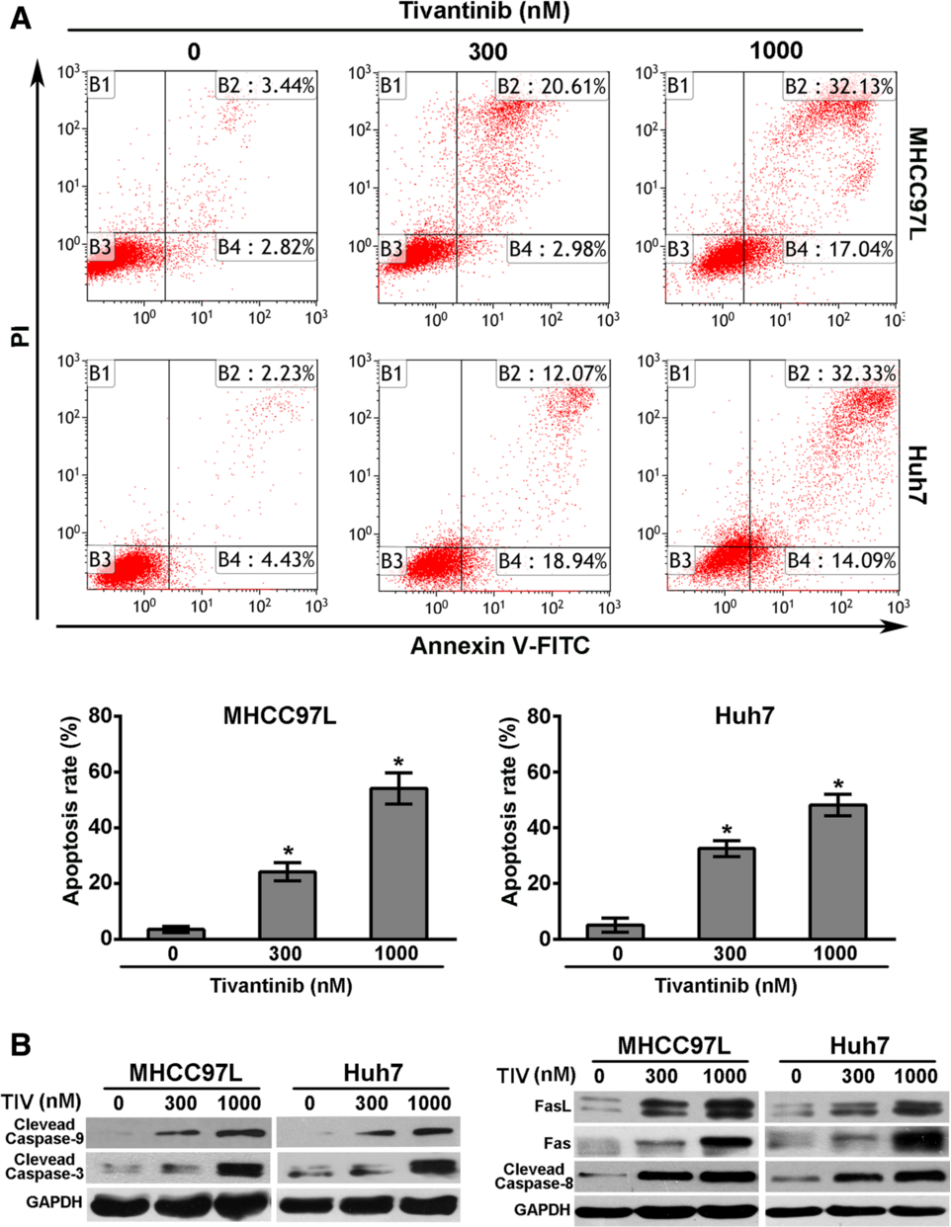
Tivantinib induces apoptosis of MHCC97L and Huh7 cells though the intrinsic and extrinsic pathway. **a** MHCC97L and Huh7 cells were treated with tivantinib for 48 h, and then stained with Annexin V–FITC and propidium iodide (PI) for flow cytometric analysis. Data were analyzed using Kaluza software and were reported as the mean ± SD. **b** Changes of the expression of apoptosis related proteins in MHCC97L and Huh7 cells treated with tivantinib for 48 h. *, *P* < 0.05 vs. the control group

Apoptosis has been proven to be responsive to tubulin-targeting agents mediated anticancer activities. Accordingly, we investigated the ability of tivantinib-induced apoptosis of MHCC97L and Huh7 cells by using flow cytometric analysis. As illustrated in Fig. 5a, the apoptotic cell population increased in MHCC97L and Huh7 cells after treatment with tivantinib for 48 h.

We were further inspired in exploring how tivantinib is involved in the activation of apoptosis. Caspase-9 is the major caspase member mediated intrinsic apopstosis, while caspase-3 is one of the key effector in downstream execution apoptotic pathway [38]. After treatment with tivantinib for 48 h, up-regulated cleaved caspase-9 and caspase-3 levels were observed in MHCC97L and Huh7 cells (Fig. 5b). Furthermore, Bcl-2 family proteins including pro-apoptotic protein Bax, and anti-apoptotic protein Bcl-2 were also investigated. Our results showed that tivantinib treatment significantly reduced the expression of Bcl-2 and enhanced the expression of Bax (data not show). Fas is the well-known member of the death receptor protein, while caspase-8 is the crucial initiator caspase in signal transmission by death receptor pathway [38]. As shown in Fig. 5b, tivantinib significant increased the expression of Fas, FasL and cleaved caspase-8. These results suggest that the apoptosis induced by tivantinib was through the intrinsic and extrinsic pathway in MHCC97L and Huh7 cells.

### Tivantinib abrogates tumor growth in MHCC97L xenograft mouse model independent of inhibition MET

**Fig. 6:**
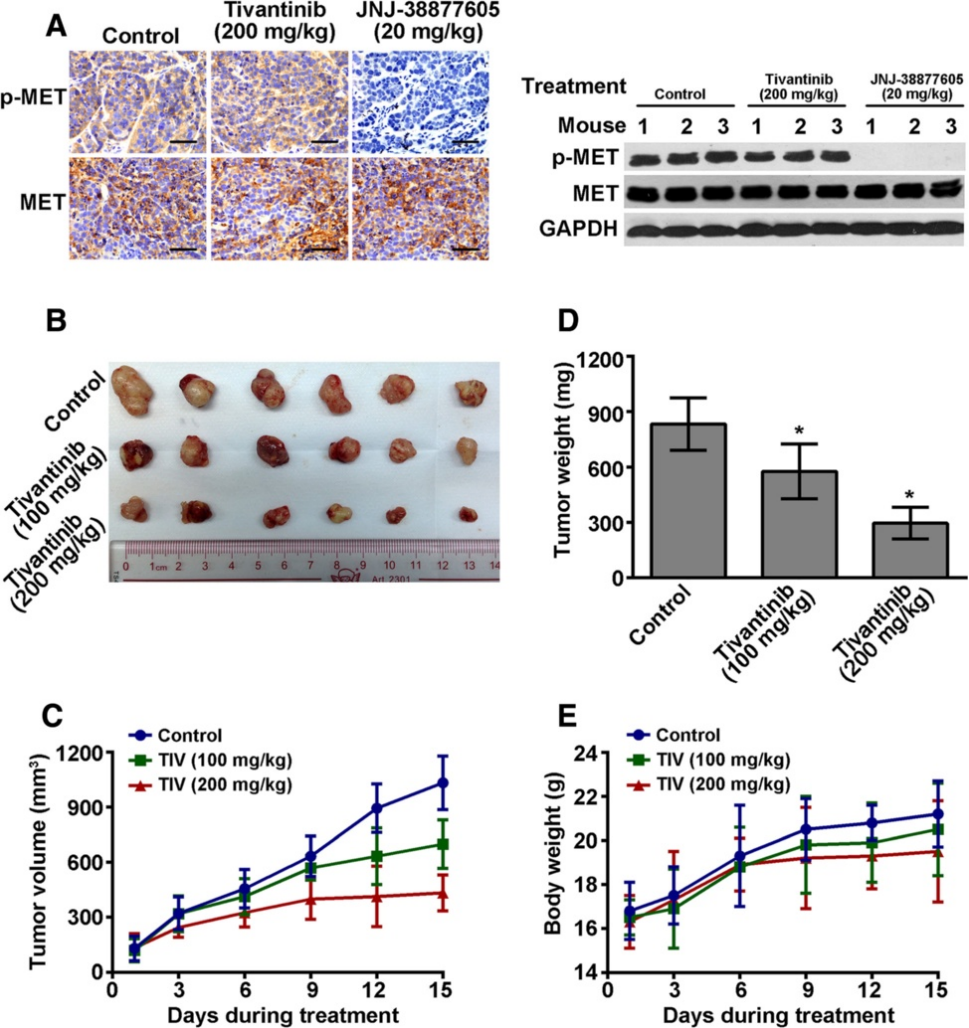
Tivantinib abrogates tumor growth in MHCC97L xenograft mouse model independent of inhibition MET. **a** Tivantinib does not inhibit phosphorylation of MET in MHCC97L tumors demonstrated by immunohistochemical and Western blot analyses. **b** The picture of the excised MHCC97L tumor from mice receiving different concentration of tivantinib treatment. **c** The changes in tumor volume over the time course of the experiment in MHCC97L xenograft model are shown. Each point on the line graph represents the mean tumor volume (mm^3^) on the corresponding day over the time course of the experiment. **d** The mean of tumor weights of the excised MHCC97L tumor from different mice. Each column represents the mean of the determinations. **e** Changes in the mean of body weight over the time course of the experiment in MHCC97L xenograft model are shown. Each point on the line graph represents the mean of the body weight on a particular day. Scale bars, 100 μm. *, *P* < 0.05 vs. the control group

To examine that tivantinib inhibits MET activity *in vivo*, established MHCC97H xenografts (*n* = 3/group) were treated daily with an oral dose of tivantinib at 200 mg/kg for 3 days. As shown in Fig. 6a, administration of tivantinib did not affect the expression of MET and p-MET tested by immunohistochemical and Western blot analysis.

**Fig. 7:**
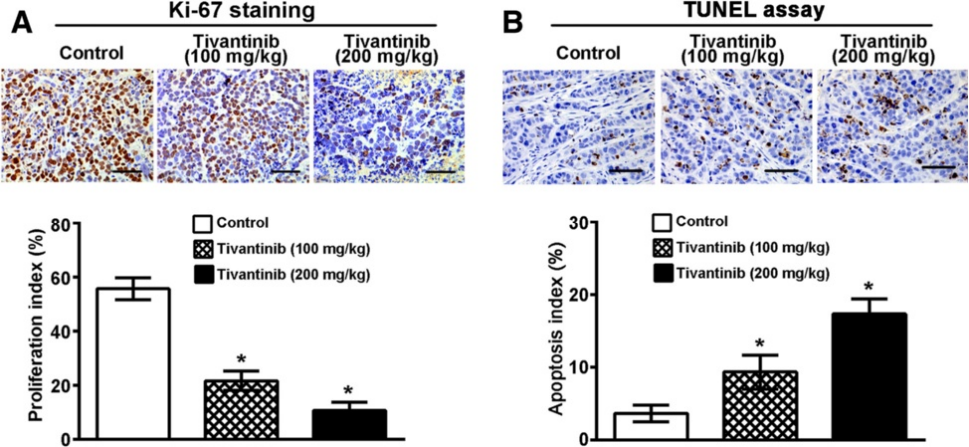
Effects of tivantinib on cell proliferation and apoptosis of MHCC97H xenografts. Mice bearing tumor xenografts were treated as described in Materials and Methods. **a** Proliferative cells stained with anti-Ki-67 antibody in tumors. **b** Apoptotic cells determined by TUNEL assay in tumors. The bar graph, mean ± SD of quantification of Ki-67 and TUNEL-labelled positive cells from analysis of tumors. Scale bars, 100. *, *P* < 0.05

To determine that tivantinib anti-tumor activity *in vivo*, established MHCC97L xenografts (*n* = 3/group) were treated daily with an oral dose of tivantinib at 100 mg/kg or 200 mg/kg for 15 days. The results in Fig. 6b, c and d demonstrated that tivantinib at concentrations of both 100 mg/kg and 200 mg/kg displayed a good anti-tumor effect on MHCC97L xenografts; their tumor growth inhibition (TGI) rates were 30.9 % and 64.6 %, respectively. Tivantinib treatment was well tolerated, as determined by stable body weights throughout the treatment period (Fig. 6e). We next evaluated the anti-proliferative and pro-apoptotic effects of tivantinib in treated tumor xenografts. Immunohistochemical analyses revealed that tivantinib decreased the expression of Ki-67 in MHCC97L xenografts by 61.1 % and 80.8 % at doses of 100 mg/kg and 200 mg/kg, respectively, compared with the control group (Fig. 7a). TUNEL assay demonstrated that the apoptotic cells in MHCC97L xenografts were increased by −60.7 % and 78.8 % at doses of 100 mg/kg and 200 mg/kg, respectively (Fig. 7b).

## Discussion

Tivantinib is being currently evaluated in the phase III clinical trial as a specific MET inhibitor in HCC. However, the relationship between clinical efficacy of tivantinib on HCC and its mechanism of action has not been established. To study this problem, we examined the effect and action of tivantinib on HCC cells *in vitro* and *in vivo*.

Our previous work have showed that inhibition of MET signalling is causally associated to anti-proliferation, only in HCC cell lines with expression of constitutive p-MET [12]. Thus, we compared the tivantinib sensitivity between MET-dependent and MET-independent HCC cell lines to investigate the mechanism underlying how tivantinib affects HCC cells growth. The results showed that the IC_50_ values of tivantinib in MET-independent Huh7 and HepG2 cells were similar to those in MET-dependent MHCC97L and HCC97H cells. Furthermore, tivantinib did not inhibit constitutive and HGF-induced p-MET in HCC cell lines examined, even though the drug concentrations inducing significant suppression of growth in these cells. Consistent with the *in vitro* study, tivantinib did not show any anti-MET activity in MHCC97H xenografts model. The data presented above collectively suggest that the anti-proliferative activity of tivantinib is not due to its effects on MET. The results of immunohistochmistry and microtubule polymerization analysis were consistent with the works led by Basilico and Katayama, indicating growth inhibition of tivantinib in HCC cells may be due to inhibition of tubulin polymerization. Furthermore, the potent anti-proliferative activity of tivantinib in MDR cells suggests it a more promising microtubule inhibitor than paclitaxel and vincristine.

Tivantinib has been shown to induce cell cycle arrest and promote apoptosis in a variety of cancer cells, including lung cancer, colorectal adenocarcinoma, gastric cancer and breast cancer [21]. However, direct anti-tumor effects of tivantinib in the treatment of HCC have not been clearly established. Cell cycle analysis examined by flow cytometric showed that treatment with tivantinib leads to the accumulation of MHCC97L and Huh7 cells in G2/M phase. It is well-demonstrated that different classes of cyclins and their cyclin-dependent kinases control cell cycle progression. In eukaryotic cells, entry into mitosis is controlled by the activation of the cyclin B/Cdc2 protein kinase, resulting in the degradation of cyclin B. The cyclin B/Cdc2 complex is activated by dephosphorylation of key residues by the Cdc25 family of phosphatases [35]. Our western blot results demonstrated that cells treated with tivantinib result in down-regulation of both cyclin B1 and Cdc25C expression and up-regulation the expression of cyclin-dependent kinase inhibitor p21, providing an explanation for the observed cell-cycle inhibition. Induction of apoptosis have been proven to contribute to the potent anti-tumor effects of the tivantinib [21]. To date, research indicates that there are two main apoptotic pathways: the extrinsic or death receptor pathway and the intrinsic or mitochondrial pathway [38]. The extrinsic signaling pathways that initiate apoptosis involve death receptors (e.g. Fas) on the cell surface. Interaction of a death receptor with its ligand triggers the formation of a death-inducing signaling complex, which in turn recruits caspase-8 [39]. The intrinsic signaling pathways that initiate apoptosis involve loss of membrane potential in mitochondria and then the release of cytochrome c from the mitochondria into the cytosol following activation of caspase-9. Both initiator caspases of the intrinsic and extrinsic lead to their own auto-activation which further activates caspase 3, the effector caspase [40]. Here, we detected the effect of tivantinib on intrinsic and extrinsic apoptosis pathway. We found apoptosis of MHCC97L and Huh7 cells induced by tivantinib were dependent on the activation of caspase-9 and FasL/Fas mediated pathway. It suggests that tivantinib induced apoptosis may be intrinsic and extrinsic apoptosis pathway dependent. Members of the Bcl-2 family can form homo- or hetero-dimers, thereby functioning as agonists or antagonists of each other [41]. Importantly, changes the ratio of pro-apoptosis Bcl-2 protein and anti-apoptosis Bcl-2 protein refer to chemotherapy resistance in many cancers [42]. Our data showed that tivantinib up-regulated the expression of Bax and down-regulated the expression of Bcl-2. Thus, the alteration of the ratio of pro-apoptotic and anti-apoptotic Bcl-2 family members may be another important mechanism for the effect of tivantinib on apoptosis of MHCC97L and Huh7 cells. The effect of tivantinib on inducing cell cycle arrest and promoting apoptosis are supported by the results of *in vivo* experiment which showed a good anti-tumor growth with anti-proliferative and pro-apoptotic effects in MHCC97L xenografts.

## Conclusions

Our study reveal that tivantinib displays cytotoxic activity in HCC cells via disrupting tubulin polymerization, consequently causing cell cycle arrest and inducing apoptosis. These results indicate that this promising and well-tolerated cytotoxic drug should not be continued to evaluated as a selective MET inhibitor for the treatment of HCC.

## Abbreviations

HCC: Hepatocellular carcinoma
RTK: Tyrosine kinase receptor
HGF: Hepatocyte growth factor
p-MET: Phosphorylation of MET
MAPK: Mitogen-activated protein kinase
PI3K: Phosphatidylinositol 3-kinase
FAK: Focal adhesion kinase
PFS: Progression-free survival
OS: Overall survival
GSK3: Glycogen synthase kinase 3
NSCLC: Non-small cell lung cancer
Dox: Doxorubicin
siRNA: Small interfering RNA
P-gp: P-glycoprotein
MDR: Multidrug resistance
TGI: Tumor growth inhibition
